# Effect of massage therapy on infants with congenital muscular torticollis: A retrospective comparative study

**DOI:** 10.3389/fped.2022.984675

**Published:** 2023-01-09

**Authors:** Wei Tang, Zhoujin Li, Weihui Xu, Yong Ye, Huijuan Wang, Ying Wang, Xiangning Shao, Mengqing Wang, Jianda Xu

**Affiliations:** ^1^ Department of Acupuncture, Moxibustion and Tuina, The First Hospital of Hunan University of Chinese Medicine, Changsha, China; ^2^ Department of Orthopaedics, The Second Affiliated Hospital of Hunan University of Chinese Medicine, Changsha, China; ^3^ Department of Acupuncture, Moxibustion and Tuina, The First Affiliated Hospital of Hunan College of Traditional Chinese Medicine, Zhuzhou, China; ^4^ Department of Orthopaedics, Changzhou Traditional Chinese Medicine Hospital, Changzhou, China

**Keywords:** infants, congenital muscular torticollis, safety, effect, massage

## Abstract

**Objective:**

To detect the effect and safety of massage therapy on infants with congenital muscular torticollis.

**Methods:**

A total of 56 infants with unilateral congenital muscular torticollis were enrolled in this retrospective comparative study. The subjects were divided in two groups, namely, the control group and the massage group. The control group (*n* = 28) received the treatment of sternocleidomastoid muscle (SCM) stretching, while the massage group (*n* = 28) received massage therapy combined with SCM stretching. The following parameters were compared: the cervical range of motion (ROM) and functional level (muscle function scale and ratio of muscle function scale scores). Complications, if any, were also recorded.

**Results:**

Of the 56 infants, 7 infants (12.5%) underwent surgery with little functional improvement. The total effective rate of conservative treatment was 87.5%. No significance was found in terms of the surgery rate between both groups (14.29 vs. 10.71%, *P* = 0.693). After treatment, the ROM (including rotation and lateral flexion) and the ratio of muscle function scale scores improved significantly (*P* < 0.05). In the latest follow-up, the massage group showed a greater improvement in rotation and lateral flexion. However, no significant difference in the muscle function scale score ratio was found (*P* = 0.126). Importantly, no adverse events related to blood vessels, nerves, and SCM occurred.

**Conclusions:**

Providing massage therapy in infants with congenital muscular torticollis is a safe and effective method to improve the cervical range of motion and function. However, this study did not find any decrease in the surgical rate between two groups of patients despite adding such therapy.

## Introduction

Congenital muscular torticollis (CMT) is a common musculoskeletal disease after birth, which occurs as a result of unilateral pathological shortening of the sternocleidomastoid muscle (SCM). The incidence varies from 0.3% to 2% (1). If not treated, it may result in clinical symptoms such as head tilt, facial asymmetry, and cervical spine dysmorphism (2). To date, the exact etiology of CMT remains unclear (3). The main potential causes include prenatal factors (i.e., maneuvers performed during delivery, intrauterine postural abnormality), SCM muscle fibrosis (infectious myositis, peripartum bleeding), and primary myopathy. Physiotherapy management has been listed as the first-line management strategy for CMT in clinical practice guidelines (4, 5).

As an integral part of traditional Chinese medicine, massage therapy is a complementary medicine treatment modality. The addition of massage therapy to the treatment protocol has been recognized as a potential treatment method for CMT. However, to our knowledge, massage therapy remains controversial because of little documentation from scientific research. The effect of massage therapy is still poorly documented.

In this study, we conducted a retrospective comparative study to evaluate whether massage therapy was tolerated and beneficial for infants with CMT. In addition, we examined the safety of massage therapy by monitoring for adverse events.

## Patients and methods

This clinical trial was designed and approved by the Ethics Review Committee of the first hospital of Hunan University of Chinese Medicine. Written informed consent from the parents of infants was obtained.

Between June 2015 and Dec. 2018, 56 infants with unilateral CMT, with an age range of 30–170 (average, 52.71 ± 52.01) days, were enrolled in this retrospective comparative study (Table 1). All infants were term deliveries and had a mass in the SCM. According to the therapeutic choice, the cohort was divided into the massage group (*n* = 28) and the control group (*n* = 28). The control group received the treatment of SCM stretching, while the massage group received massage therapy combined with SCM stretching. At baseline, no significant difference (*P* > 0.05, Table 1) was found in terms of age, gender, laterality, birth weight, delivery type, cervical lateral flexion of the affected side, cervical rotation of the affected side, degrees, and muscle function scale score ratio (*P* > 0.05, Table 1).

**Table 1:** The baseline of all infants with SCM.

Variable	Control group	Manual group	*P-*value
Age, *d*	46.89 ± 36.52	43.46 ± 33.90	0.598
Gender, male/female, *n*	20/8	19/9	0.771
Laterality, left/right, *n*	11/17	17/11	0.181
Birth weight (*g*)	3102.00 ± 187.02	3076.00 ± 152.86	0.448
Delivery type (NSVD/C-sec)	22/6	21/7	0.752
Cervical lateral flexion of the affected side, degrees	47.31 (11.07)	45.00 (10.11)	0.45
Cervical rotation of the affected side, degrees	47.25 ± 4.86	44.00 ± 3.61	0.583
Muscle function scale score ratio (affected side/unaffected side)	1.80 (0.68)	1.56 (0.86)	0.386

NSVD, normal spontaneous vaginal delivery; C-sec, cesarean section.

The exclusion criteria applied were bilateral SCM, any specific anomaly that could affect the cervical range of motion (ROM), any health problems preventing the performance of the intervention or assessment, and the unwillingness of the parents of infants to participate in the study.

## Physiotherapy regimen

Massage therapy and stretching were performed by the same experienced pediatric outpatient physiotherapist who was a major in massage.

## Control group

### SCM stretching

According to the guidelines of CMT (4), pain-free stretching is advocated to prevent micro injuries to the SCM muscle. It is the most typical and safe choice to improve the extensibility of the SCM and decrease the surgical rate. Usually, it is combined with handling and positioning to improve postural alignment.

In this study, the frequency of therapy was about three times a week and adjusted by initial local condition assessment. The stretching protocol was as follows: intensity (10 stretches a session), sustained (5–10 s per stretching on the tolerance level), rest interval (10 s between any 2 stretches), and frequent (5 sessions per day).

In this study, all infants received the two-person stretching treatment with the supine position. One person stabilized the shoulders of the infant, while the other stood above the head and stretched the SCM through the available range of neck. In this technique, attention should be paid to the range of rotation (<90°) for ensuring sufficient blood flow.

## Massage group

Before SCM stretching, massage therapy was additionally introduced with the same level of frequency.

Based on conservative treatment protocol and our own experience, we advocated massage therapy (finger rubbing and stroking method) before SCM stretching. Its aim was to reduce tension and make the SCM muscle more flexible. To achieve a consistent level of intensity, the massage therapy was performed by the same senior physician.

Finger rubbing and stroking method: the physician rubbed the SCM muscle with the index finger, middle finger, and ring finger for 3 min and then gently stroked the SCM muscle longitudinally with the thumb from the starting point to the ending point of the muscle for 2–3 min. Here, one should be careful not to press the larynx and the vascular bundle of the neck.

### Clinical parameters

The outcome was measured by using the cervical ROM (lateral flexion and cervical rotation) and muscle function scale. The follow-up duration is defined as the time between the initial treatment and the current outpatient treatment time. Here, the follow-up duration of all infants was 12.29 ± 5.12 (range, 6–23) months. Surgery was advised if passive neck rotation was >6° or lateral flexion was >6° after therapy.

### Cervical ROM

Based on the method previously confirmed (6), a special large arthrodial protractor was used to measure the head tilt and the ROM (degrees of lateral flexion and rotation) when the infants lay in the supine position. Head tilt is defined as the angle between the midline of the head and the midline of the body.

### Functional assessment

The muscle function scale (7) was employed to assess the muscle function of both sides. The scores varied from 0 to 5 but recorded from 1 to 6 for subsequent statistical analyses.

The ratio of muscle function scale scores = muscle function scale score of the affected side/muscle function scale score of the unaffected side.

### Complications

Complications, if any, were also recorded.

### Statistical analysis

Data were analyzed employing SPSS for Windows version 24.0 (SPSS Inc., Chicago, IL, United States). The normality of distribution for continuous numeric variables was assessed using the Kolmogorov–Smirnov test. Normally distributed variables were presented as means with SDs. The characteristics of the two groups were analyzed by using Student’s *t*-test, the chi-square test, or the Wilcoxon Rank Sum test. ROM were compared by using repeated measures ANOVA. Statistical significance was set at *p* < 0.05.

## Results

Of the 56 infants, 7 (12.5%) underwent surgery with little functional improvement. The total effective rate of conservative treatment was 87.5%. No significance was found in terms of the surgical rate (14.29% vs. 10.71%, *P* = 0.693).

### Cervical range of motion

The ROM, including rotation and lateral flexion, was smaller on the affected side. Both lateral flexion and rotation improved significantly after treatment (*P* < 0.001, Table 2). Compared with the control group, the massage group showed a greater improvement in rotation (80.68 ± 11.66 vs. 65.32 ± 12.57, *P* = 0.046, Table 2) and lateral flexion (52.00 ± 2.64 vs. 49.39 ± 4.25, *P* = 0.040, Table 2) as indicated by the independent-samples *t*-test.

**Table 2:** Changes in the cervical range of motion.

	Time	Control group	Manual group	*P*-value
Cervical rotation ROM (°)	Initial	47.25 ± 4.86	44.00 ± 3.61	0.583
	3 months later	65.32 ± 12.57	80.68 ± 11.66	0.046
	*P*-value	0.000	0.000	
Cervical lateral flexion ROM (°)	Initial	26.25 ± 5.91	26.33 ± 7.51	0.521
	3 months later	49.39 ± 4.25	52.00 ± 2.64	0.040
	*P*-value	0.000	0.000	

ROM, range of motion.

### Changes in the ratio of muscle function scale scores

In the baseline of both groups, no significant difference was found in the ratio of muscle function scale scores (2.17 ± 0.72 vs. 2.05 ± 0.81, *P* = 0.231, Table 3). Also, the ratio of muscle function scale scores in both groups improved significantly after treatment (*P* = 0.002 and 0.001, Table 3). However, no significant difference in this ratio in these groups was found after treatment (-0.12 ± 0.60 vs. −0.19 ± 0.65, *P* = 0.126, Table 3).

**Table 3:** Changes in the ratio of muscle function scale scores.

	Time	Control group	Manual group	*P*-value
Muscle function scale score ratio (affected side/unaffected side)	Initial	2.17 ± 0.72	2.05 ± 0.81	0.231
	3 months later	−0.12 ± 0.60	−0.19 ± 0.65	0.126
	*P*-value	0.002	0.001	

### Complications

No adverse events related to blood vessels, nerves, and the SCM muscle occurred.

### Discussion

In this study, our findings confirmed that providing massage therapy in infants with CMT was a safe and effective method to improve the cervical range of motion and function. However, no decrease in the surgical rate was found despite such therapy.

CMT is a common musculoskeletal disease after birth and is a curable childhood condition. Good results can be achieved if the disease is diagnosed early and suitable treatment is provided. The earlier the initiation of therapeutic procedures, the shorter the treatment duration. In this study, most infants received treatment for less than 3 months. The total effective rate of conservative treatment was 87.5%.

Physical therapy is used to effectively improve passive cervical ROM and function and not just to reduce the mass. To date, little is known about the efficacy of ultrasonography findings in clinical prognosis (8). Hwang et al. confirmed that 3 months of physical therapy could reduce the physical stiffness of the fibrotic mass and restore the cervical ROM in infants with CMT (9). Poole and Kale reviewed the literature published from 2011 to 2019 and pointed out that stretching of the affected SCM was an effective conservative physiotherapy method for infants with CMT. The earlier the referral, the lesser the treatment duration (10). It was confirmed that SCM stretching could effectively elongate and increase muscular elasticity (11). The SCM was deliberately extended to improve muscle flexibility and elasticity. This procedure stimulated the repair and normal myofibrils of an abnormal SCM (5). Our results also showed that the ROM (including rotation and lateral flexion) and the ratio of muscle function scale scores improved significantly after treatment (*P* < 0.05), which was consistent with the previous results. One research was performed to investigate the effect of the different dosages of stretching treatment on the prognosis of infants with CMT. The researchers found that stretching for 100 times brought about better muscle function and range of motion than for 50 times a day (11). Did this mean that the more the stretching times, the better? Further research was needed to identify the appropriate level of intervention dosage.

As an integral part of traditional Chinese medicine, massage therapy is a complementary medicine treatment modality. Massage therapy can convert mechanical energy into heat energy, accelerate blood circulation, promote metabolism, and eliminate the adhesion between the mass and the sternocleidomastoid muscle. The results in this study confirmed that the massage group showed a greater improvement in rotation and lateral flexion at the latest follow-up; however, no significance was found in terms of the surgery rate, as mentioned previously (14.29 vs. 10.71%, *P* = 0.693). However, Haugen et al. conducted a randomized double-blinded controlled trial and found that the addition of manual therapy in the form of massage to the treatment protocol did not provide a better short-term effect than physiotherapy alone (12). More prospective studies with a big sample size are needed to buttress this finding.

Muscle function scale score ratio is a valid tool to evaluate the imbalance in muscle function around the neck. A previous study also found that SCM muscle strength seemed to improve significantly after stretching treatment (6). Ultrasonographic examination revealed that stretching promoted the growth of bilateral SCM and helped achieve a better balance of SCM thickness (11). After treatment, the ratio of muscle function scale scores improved significantly (*P* < 0.05). However, no significant difference in this ratio was found (*P* = 0.126). A similar conclusion was not drawn based on our study.

Many studies are performed to determine prognostic risk factors for infants with CMT. The degree of SCM injury and the time of initial physiotherapy have been found to be related to the resolution of CMT. Hsu et al. found that clinical severity was strongly correlated with sonographic features. SCM thickness denoted the degree of proliferation of fibrous tissue (13). Ryu et al. used ultrasonography to evaluate the potential factors influencing the regression of infants with CMT. A percentage of 79.4 infants undergoing physiotherapy underwent a complete restoration of SCM thickness. Physical therapy was regarded as the only statistically significant factor influencing the resolution of CMT symptoms (14). They also found that the age at diagnosis was independently associated with the restoration of SCM thickness. Early referral was encouraged for achieving a successful treatment outcome.

Infants with CMT can recover completely without any functional residuals if treated early and appropriately. A mass on the lateral side of the neck can be detected upon physical examination up to 3 months of birth. Early diagnosis and immediate correction are recommended to avoid the regression and fibrous contraction of mass (15). Previous research also concluded the same results and pointed out that early referral to physical therapy could help shorten the treatment duration (16). The results revealed that children with CMT treated before 1 month of life showed better improvement than those treated at 3–4 months of life (98% vs. 85) (17). Moreover, treatment before 6 weeks could improve the range of motion significantly (18).

### Limitations

This study had some limitations. First, it was a retrospective study designed with a small sample size. Second, the follow-up time was 12.29 ± 5.12 (range, 6–23) months, which may not reflect the possible long-term effects of massage therapy. Third, the enrolled patients did not represent the whole spectrum of infants with CMT, because all infants were term deliveries and had a mass in the SCM without any morphological changes, as shown by an ultrasonographic examination. And the infants came from just one single center, multicenter studies are needed.

## Conclusion

Providing massage therapy in infants with congenital muscular torticollis is a safe and effective method to improve the cervical range of motion and function. However, adding such therapy to the overall treatment did not result in a decrease in the surgical rate, as found in the present study. Therefore, further prospective controlled trials are necessary for providing better treatment recommendations.

## Data Availability

The raw data supporting the conclusions of this article will be made available by the authors, without undue reservation.
